# Pacemaker Placement in Patients with Stroke-Mediated Autonomic Dysregulation

**DOI:** 10.1155/2017/6301430

**Published:** 2017-03-16

**Authors:** Ali A. Alsaad, Christopher O. Austin, Maisha T. Robinson, Michael B. Phillips

**Affiliations:** ^1^Department of Internal Medicine, Mayo Clinic, Jacksonville, FL 32224, USA; ^2^Division of Cardiovascular Diseases, Mayo Clinic, Jacksonville, FL 32224, USA; ^3^Department of Neurology, Mayo Clinic, Jacksonville, FL 32224, USA

## Abstract

Lateral medullary syndrome (LMS) is an ischemic disease of the medulla oblongata, which involves the territory of the posterior inferior cerebellar artery. Lateral medullary syndrome is often missed as the cause of autonomic dysregulation in patients with recent brain stem stroke. Due to the location of the baroreceptor regulatory center in the lateral medulla oblongata, patients with LMS occasionally have autonomic dysregulation-associated clinical manifestations. We report a case of LMS-associated autonomic dysregulation. The case presented as sinus arrest and syncope, requiring permanent pacemaker placement. A dual-chamber pacemaker was placed, after failure of conservative measures to alleviate the patient's symptoms. Our case shows the importance of recognizing LMS as a potential cause for life-threatening arrhythmias, heart block, and symptomatic bradycardia. Placement of permanent pacemaker may be necessary in some patients with LMS presenting with syncope, secondary to sinus arrest.

## 1. Introduction

Lateral medullary syndrome, also known as Wallenberg's syndrome, is an ischemic disease of the medulla which involves the territory of the posterior inferior cerebellar artery [1]. The clinical triad typically consists of contralateral sensory deficit, ipsilateral ataxia, and ipsilateral Horner's syndrome. Due to the location of the baroreceptor regulatory center in the lateral medulla oblongata, patients with Wallenberg's syndrome occasionally have autonomic dysregulation [2]. Infarction of this intricate territory can lead to lability of vascular tone and heart rate, resulting in recurrent syncope [1, 3]. We report a case of LMS with autonomic dysregulation in a patient with sinus arrest and syncope, treated successfully by permanent pacemaker placement.

## 2. Case Report

**Figure 1:**
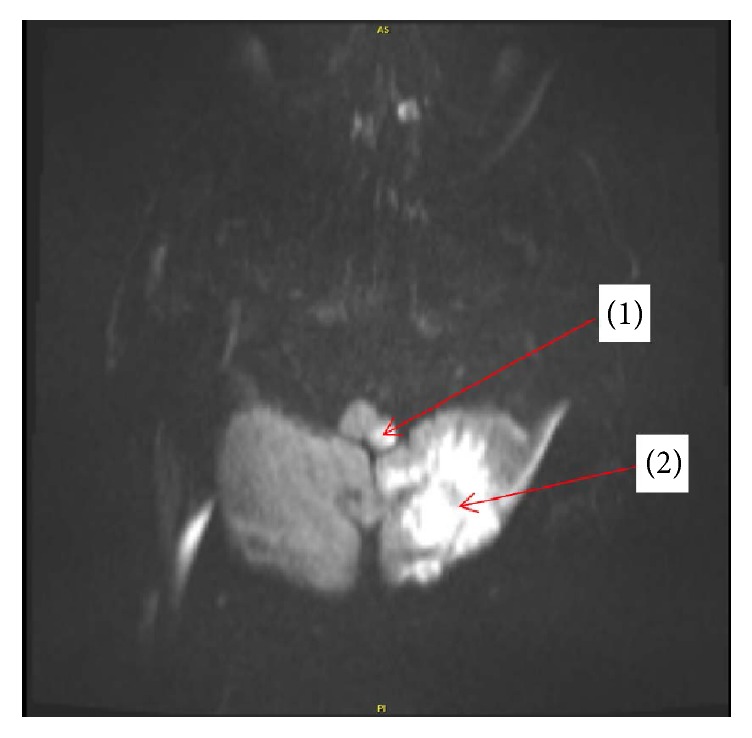
Axial diffusion weighted imaging sequence of a magnetic resonance imaging scan of the brain demonstrates hyperintensity in various brain areas. Those findings are consistent with an acute stroke in the left medullary restiform body (1) and left cerebellar hemisphere (2).

An 85-year-old man, with a history of normal pressure hydrocephalus, treated with ventriculoperitoneal shunt, coronary artery disease, hypertension, and mild dementia, presented with severe, intractable nausea, vomiting, and hoarseness associated with ataxia and recurrent syncopal episodes. Magnetic resonance imaging (Figure 1) revealed an acute infarction in the left cerebellar tonsil, left cerebellar hemisphere, and left medullary restiform body, in the territory of the left posterior inferior cerebellar artery (PICA). Radiologic findings were consistent with lateral medullary infarction. The clinical picture was suggestive of lateral medullary syndrome (LMS).

The exact reason for the brainstem and cerebellar stroke remained unclear, as diagnostic testing including transesophageal echocardiography and carotid ultrasound failed to reveal any significant abnormalities. Also, cardiac telemetry monitoring did not reveal any evidence of supraventricular arrhythmias, including atrial fibrillation.

During hospitalization, the patient experienced multiple syncopal episodes. The syncopal episodes were brief and predominately associated with postural changes, as well as significant nausea and vomiting. No seizure-like activity was noted and both the patient and his caregiver denied any history of similar episodes of nausea, vomiting, or syncope prior to this admission.

**Figure 2:**
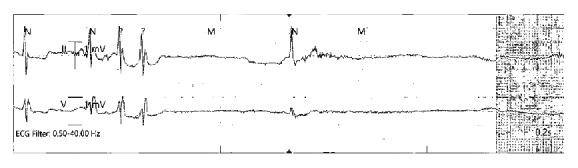
Telemetry strip during one of the syncope episodes showing a junctional escape beat overlaying a sinus arrest (6-second pause) proceeded by premature atrial contractures.

During the events, the patient was witnessed to have sinus arrest, lasting up to 6 seconds, with an intermittent junctional escape rhythm (Figure 2). Otherwise, the baseline electrocardiography and cardiac telemetry was sinus rhythm, with heart rate 50–60 beats per minute and right bundle branch block. The patient had documented blood pressure lability during the syncopal episodes, with a peak of 218/92 mm Hg. This represented a substantial difference from prior baseline blood pressure of 130/70 mm Hg. Physical examination was remarkable for cerebellar ataxia and nystagmus. No sensory loss or signs of Horner syndrome were found to suggest a full constellation of lateral medullary syndrome. No significant abnormalities were found in laboratory tests results. The patient's medication regimen at the time of the initial events included metoprolol, bethanechol, and clonidine, all of which possess potential atrioventricular nodal blocking properties. Therefore, all of those medications were discontinued. Lisinopril was administered daily to control hypertension.

Functional assessment of the Codman® Hakim® shunt (Codman/Johnson & Johnson, Raynham, Massachusetts) by fluoroscopic shunt evaluation revealed a pressure of 140 cm H_2_O from the last recorded setting, which was 130 cm H_2_O. However, adjustment of the shunt settings to 130 cm H_2_O failed to control his autonomic symptoms or prevent recurrent syncope and associated sinus arrest. This finding excluded Cushing reflex as an etiology for symptomatic bradycardia, secondary to elevated intracranial pressure.

The patient's symptoms continued to occur over the course of the next few days, despite the discontinuation of all potential medications with chronotropic and/or dromotropic effects. Five days after the initial symptoms and due to these persistent events, the patient underwent placement of a dual-chamber pacemaker. An L101 Essentio® pacemaker (Boston Scientific, Marlborough, Massachusetts) was placed with two leads, to right atrium and right ventricle with DDD mode, with resolution of syncopal events. However, his nausea and vomiting persisted. The patient was subsequently discharged to poststroke rehabilitation center, with prescribed antiemetic medications to control nausea and vomiting.

Follow-up at 1, 3, and 6 months revealed that the patient had no syncopal or presyncope episodes after the placement of the pacemaker. Interrogation of the pacemaker revealed normal device and lead function with no recorded arrhythmia.

## 3. Discussion

Lateral medullary, or Wallenberg's, syndrome was originally described by Adolf Wallenberg more than 100 years ago. It was described as a constellation of signs and symptoms, including nausea, vomiting, hoarseness, ataxia, vertigo, and sensory loss secondary to lateral medullary infarction [1, 2]. Horner syndrome of unilateral miosis, ptosis, and anhidrosis may also be seen. Dysphonia and hoarseness are well described in LMS and they were present in our patient. The mechanism of the stroke in LMS is usually atherosclerotic, secondary to plaque rupture in the vertebral artery or the posterior inferior cerebellar artery. Cardiac and carotid emboli are also potential mechanisms for this disease. Rarely, LMS can be caused by vertebral artery dissection, involving the distribution of posterior inferior cerebellar artery [3]. Anatomically, the medulla encompasses the baroreceptor regulatory center and its impairment may result in labile blood pressures and bradycardia [1, 4]. Autonomic dysregulation may be secondary to the involvement of several medullary nuclei, including nucleus tractus solitarius and nucleus ambiguus, which aid in regulation of autonomic function [4–6].

Previous case reports have described autonomic dysfunction in patients with medullary infarctions. Hong et al. [4] reported cases of autonomic dysfunction, specifically parasympathetic, in patients with infarctions of the ventrolateral right medulla [6]. Sinus arrest, requiring placement of permanent cardiac pacemaker, has also been described in the literature in association with LMS [7, 8].

Cushing reflex, the classic triad of systemic hypertension, bradycardia, and increased intracranial pressure, would be a plausible explanation for our patient's presentation. However, no symptomatic benefit was achieved after adjustment of intracranial pressure using the Codman Hakim system, making this an unlikely explanation for the patient's presentation.

Although our patient did not have prototypical LMS, with associated Horner syndrome and sensory loss, he did have clear evidence of lateral medullary infarction localized to the cerebellum and inferior cerebellar peduncle (restiform body) on MRI. More importantly, our patient had persistent syncope, ataxia, and nystagmus, despite appropriate medical therapy, secondary to parasympathetic tone dysregulation from lateral medullary infarction. Fortunately, the syncopal episodes responded to permanent cardiac pacing, supporting the hypothesis that syncope was predominately the result of sinus arrest (cardioinhibitory response), as opposed to dysautonomia-associated hypotension (vasodepressor response), for which the role of cardiac pacing is poorly defined.

It is important to note that the patient had no prior history of stroke and his MRI indicated no areas of old infarctions. In particular, insular and hypothalamic regions appeared to be free of acute and previous strokes on MRI. Those areas are especially known to be associated with autonomic dysregulation [9, 10]. This point suggests a different localization of the stroke-mediated autonomic dysregulation in our index case, specifically the medullary restiform body and left cerebellar hemisphere.

A transient episode of atrial fibrillation and sinus arrhythmia could have been the etiology for his symptoms, as well as stroke. However, there were neither reported symptoms of nausea, vomiting, or syncope, nor any episodes of supraventricular arrhythmia on continuous cardiac telemetry. Whether the stroke was cardioembolic or atherosclerotic, resolution of recurrent syncope after pacemaker placement suggests symptomatic benefit, which may be applicable in selected cases.

In conclusion, lateral medullary syndrome is a potential cause for life-threatening arrhythmias, heart block, and symptomatic bradycardia. In-hospital telemetric monitoring is recommended following stroke, with associated LMS. Placement of a permanent pacemaker may be necessary for some patients with medullary infarcts, in order to control sinus arrest secondary to dysautonomia. Based on this case experience, we recommend placing a pacemaker if no symptomatic benefit is obtained by conservative measures alone.
